# Poor sleep is highly associated with house dust mite allergic rhinitis in adults and children

**DOI:** 10.1186/s13223-017-0208-7

**Published:** 2017-08-16

**Authors:** Damien Leger, Bénédicte Bonnefoy, Bernard Pigearias, Bertrand de La Giclais, Antoine Chartier

**Affiliations:** 1 Université Paris Descartes, Sorbonne Paris Cité, APHP, Hôtel-Dieu, Centre du Sommeil et de la Vigilance, 75004 Paris, France; 2 50000 Saint-Lô, France; 3 06000 Nice, France; 4 Centre du sommeil d’Annecy-Argonay, 74370 Argonay-Annecy, France; 5 Medical Department, ALK, 92400 Courbevoie, France

**Keywords:** Allergen immunotherapy, Allergic rhinitis, House dust mite, Insomnia, Quality of life, Real-life study, Sleep disorders

## Abstract

**Background:**

Sleep disorders are often underreported to physicians by patients with allergies. This study aimed to characterize the sleep disorders associated with respiratory allergy to house dust mites (HDM) at the time of initiation of sublingual allergen immunotherapy (SLIT) in routine clinical practice.

**Methods:**

This prospective, cross-sectional, observational study was conducted between November 2014 and March 2015 at 189 French trial sites and included 1750 participants suffering from HDM allergy who were initiating SLIT. Participants aged less than 5 years old and those who had previously started an allergen immunotherapy (AIT) for HDM allergy were not enrolled in the study. Sleep disorders were assessed by self-administered questionnaires: the Epworth Sleepiness Scale (ESS), the Insomnia Severity Index (ISI) and a modified version of the Hotel Dieu-42 (HD-42) sleep disorder questionnaire. Logistic regression models adjusted for obesity, smoking status, asthma control and nasal obstruction were used to study the relationship between allergic rhinitis (AR) classification and sleep disorders/complaints.

**Results:**

Of the 1786 participants enrolled, 1750 (907 adults and 843 children) composed the analysis population. The majority of participants (73.5% of adults and 65.8% of children) reported that their sleep disorders had prompted them to consult their physician. The most commonly observed sleep complaints were poor-quality sleep (50.3% of adults and 37.3% of children), snoring (48.1 and 41.4%, respectively) and nocturnal awakening (37.6 and 28.2%, respectively). Difficulties falling asleep were reported by 27.0% of adults and 24.7% of children. Adults and children suffering from severe persistent AR experienced sleep complaints significantly more often than participants with intermittent or mild persistent AR.

**Conclusions:**

This study highlights the high frequency of sleep disorders and their significant impact on patients with AR induced by HDM, in particular when AR is persistent and severe. Consequently, asking allergic patients about the quality of their sleep appears to be important, especially when the patient has persistent and severe AR.

## Background

Allergic rhinitis (AR) is common and is estimated to affect approximately 25% of the population in France and Canada [1, 2]. Allergies affect sleep, which can lead to tiredness and other deficits in quality of life [1, 3, 4]. House dust mites (HDM) are the primary cause of respiratory allergies [5]. HDM allergens are very prevalent in the sleep environment and are very difficult to avoid; furthermore, because it is perennial and often associated with asthma, HDM allergy has a greater effect on patients’ sleep than other allergies [6]. The decision to start allergen immunotherapy (AIT) is a marker of disease severity, and thus patients at this stage of care represent a subpopulation of interest when assessing the impact of allergy on sleep. In most studies that have been conducted on this topic to date, the methods used to assess sleep have been limited, as they have primarily used Juniper’s Rhinoconjunctivitis Quality of Life Questionnaire (RQLQ) [7, 8]. Consequently, there are insufficient descriptive data regarding the exact nature and frequency of sleep disorders. In this context, this trial termed the ‘MORPHEE study’, aimed to better characterize sleep disorders in participants with respiratory allergy to HDM who were starting sublingual allergen immunotherapy (SLIT) to assess the specific unexplored medical needs of these participants in relation to sleep disorders.

## Participants and methods

### Study design

This was a prospective, cross-sectional, observational study conducted at 189 sites in France. In total, 801 investigators were randomly selected from the records obtained in the Thalès Cegedim survey database, which contains information on physicians with “allergology” activity within the following specialties: general practice, pneumology, paediatrics, ear, nose and throat (ENT) and dermatology. Randomization was performed to achieve the highest level of representativeness of the sample based on feasibility and geographic distribution (number of physicians per French Department proportional to Departmental physician density). Overall, 212 investigators (26.5%) agreed to participate in the trial. Each investigator prospectively included 6–10 participants who met the inclusion/exclusion criteria in consecutive consultations.

The study was conducted in compliance with the Declaration of Helsinki, Good Epidemiological/Pharmacoepidemiological Study guidelines, good practice guidelines and local regulations.

The study protocol was approved by the relevant French review boards “Comité Consultatif sur le Traitement de l’Information en matière de Recherche dans le domaine de la Santé” (CCTIRS) and “Commission Nationale de l’Informatique et des Libertés” (CNIL).

### Participants

Eligible participants were at least 5 years old and starting SLIT for HDM allergy. Participants who had previously started an AIT (subcutaneous or sublingual) for an HDM allergy were not enrolled in the trial. Participants were analysed according to age, i.e., 5–17 years old [children] and 18 years and more [adults]. Oral informed consent was obtained from all participants.

### Collected data

For each participant enrolled in the study, the different variables were recorded on a paper case report form (CRF). The CRF was completed by the physician himself for each participant who met the inclusion/exclusion criteria. Each participant and/or his/her legal representative (for participants or children unable to answer on their own) also completed a self-administered questionnaire. All data were collected anonymously.

### Sleep assessment

Sleep disorders were assessed by reliable self-administered questionnaires commonly used in different populations: the Epworth Sleepiness Scale (ESS), the Insomnia Severity Index (ISI) and a modified version of the Hotel Dieu-42 (HD-42) sleep disorder questionnaire. The ESS is a subjective tool constructed and tested in the early 1990s [9, 10] that has been widely used in previous studies to assess sleepiness [4]. The ISI is a reliable and valid instrument used to quantify the severity of perceived insomnia [11]. The HD-42 questionnaire is a 42-item questionnaire that has been validated in several epidemiological studies and was developed to assess the main sleep disorders according to the Diagnostic and Statistical Manual of Mental Disorders, 5th edition (DSM-V), and the International Classification of Sleep Disorders, 3rd edition (ICS-3) [12, 13].

Sleep disorders were defined using the categories of items and criteria shown in Table 1.

**Table 1:** Definition of sleep disorders

Items	Criteria
Difficulty falling asleep	The participant needs more than 30 min to fall asleep
Nocturnal awakening	The participant wakes up at least 2 times throughout the night
Early awakening	The participant wakes up very early or extremely early in the morning
Poor-quality sleep	The participant reports «sleep trouble» as a symptom that prompted him/her to visit a doctor and «poor-quality of sleep» as one of his/her sleep disorders AND/OR the participant reports being dissatisfied or very dissatisfied with his/her current sleep AND/OR the participant reports substantial difficulties staying asleep AND/OR the participant reports considerable difficulties falling back to sleep (rarely or never managing to fall back asleep)
Snoring	The participant reports snoring often or almost daily AND/OR the participant reports that he/she was already told that he/she snores.
Insomnia	No insomnia = ISI score: 0–7 Mild insomnia = ISI score: 8–14 Moderate insomnia = ISI score: 15–21 Severe insomnia = ISI score: 22–28
Daytime sleepiness	No daytime sleepiness = ESS score: 0–8 Daytime sleepiness = ESS score: 9–15 Severe daytime sleepiness = ESS score: >15

The ISI was used to assess the severity of insomnia in the past month. The 7 items on the ISI were scored by the participant on a scale ranging from 0 to 4; the scores were then summed to obtain a total insomnia score, which was presented quantitatively and by category (0–7: no insomnia, 8–14: sub-clinical insomnia (mild), 15–21: clinical insomnia (moderate), 22–28: clinical insomnia (severe).

The severity of daytime sleepiness was globally assessed using the ESS scale. The 8 items on the ESS scale were scored on a scale ranging from 0 to 3, and these scores were summed to obtain a daytime sleepiness score. The total score was presented quantitatively and by category (0–8: no daytime sleepiness, 9–15: daytime sleepiness, >15: daytime sleepiness requiring additional tests to be conducted).

### Statistics

The statistical analysis was performed using SAS version 9.2 (SAS Institute Inc. North Carolina USA).

All variables collected in the CRFs and questionnaires and the derived parameters were included in the descriptive statistical analysis. Quantitative variables were analysed in terms of the mean, standard deviation, median, first quartile, third quartile, and extreme values. Binary, categorical, and ordinal parameters were analysed regarding the number and frequency within various categories.

The primary objective of the MORPHEE study was to describe the nature and frequency of sleep disorders in participants with respiratory allergy to HDM for whom SLIT had been initiated. All data relating to quality of sleep in the participants’ self-administered questionnaire were described for the analysis population overall and for subgroups according to age (adult/child) and type of allergic rhinitis (AR) (according to the revised ARIA 2010 criteria [14]: mild intermittent/mild persistent/severe intermittent/severe persistent). Logistic regression models adjusted for body mass index (obesity or overweight vs. others), smoking status (current smoker or subject currently exposed to passive smoking vs. non-smoker or former smoker), asthma control (non controlled asthma vs. controlled asthma or no asthma) and nasal obstruction (severe or moderate vs. slight or absent) were used to study the relationship between rhinitis status (persistent severe vs. other type of rhinitis) and sleep complaints/disorders: difficulty falling asleep, nocturnal awakening, poor-quality sleep, snoring, daytime sleepiness and clinical insomnia. The results were presented as the odds ratio (OR) and 95% confidence interval (CI). The p values associated with the explanatory variable and the model variables were also presented. The quality of model adjustment was tested using the Hosmer–Lemeshow test.

## Results

From November 2014 to March 2015, 1786 participants were enrolled in the study by 189 physicians. Following verification of the inclusion/exclusion criteria, 36 participants were excluded from the analysis population, half of them due to missing data related to SLIT prescription. Thus, 1750 participants (907 adults and 843 children) were retained for the analysis.

### Participant characteristics

The main characteristics of the participants are summarized in Table 2. A large majority of the participants lived in town (71.4%) and in low-altitude locations (75.5%). Neck circumference was high for 12.8% of adults and most adults had a low risk of obstructive sleep apnea (risk score between 0 and 2) according to physician judgement based on participant interviews without polysomnography.

**Table 2:** Characteristics of participants initiating SLIT for HDM allergy

Variable	Statistics	Children n = 843	Adults n = 907	p value	Total n = 1750
Sex	*N (missing data)*	*836 (7)*	*902 (5)*		*1738 (12)*
Male	N (%)	479 (57.3)	346 (38.4)	<0.001 (S)*	825 (47.5)
Age (years)	*N*	*843*	*907*		*1738*
	Mean ± SD	10.5 ± 3.5	33.2 ± 11.7		22.3 ± 14.3
Family history of allergy	N (%)	656 (77.8)	577 (63.6)	<0.001 (S)*	1233 (70.5)
Polysensitization	N (%)	466 (55.3)	565 (62.3)	0.003 (S)*	1031 (58.9)
Contact with pets	*N (missing data)*	*805 (38)*	*874 (33)*		*1679* (*71*)
Regular contact	N (%)	421 (52.3)	420 (48.1)	0.082 (NS)*	841 (50.1)
Weight	*N (missing data)*	*812 (31)*	*900 (7)*		*1712 (38)*
Obesity	N (%)	92 (11.3)	79 (8.8)	0.079 (NS)*	171 (9.8)
Moderate or high risk of sleep apnea syndrome	N (%)	–	148 (16.3)		–
Smoking status	*N (missing data)*	*792 (51)*	*901 (6)*		*1693 (57)*
Current smoker or currently exposed to passive smoking	N (%)	88 (11.1)	119 (13.2)	0.189 (NS)*	207 (12.2)
Sports activity	*N (missing data)*	*834 (9)*	*905 (2)*		*1739 (11)*
No regular sports activity	N (%)	411 (49.3)	642 (70.9)	<0.001 (S)*	1053 (60.6)
Rhinitis assessment	*N (missing data)*	*762 (81)*	*814 (93)*		*1576 (174)*
Persistent rhinitis	N (%)	655 (86.0)	732 (89.9)	0.015 (S)*	1387 (88.0)
RTSS	Mean ± SD	9.0 ± 3.4	9.8 ± 3.6	<0.001 (S)^#^	9.5 ± 3.5
TNSS	Mean ± SD	7.3 ± 2.4	7.7 ± 2.4	<0.001 (S)^#^	7.5 ± 2.4
Asthma assessment	*N*	*843*	*907*		*1750*
Associated asthma	N (%)	407 (48.3)	329 (36.3)	<0.001 (S)**	736 (42.1)
Persistent asthma	N (%)	211 (25.0)	158 (17.4)	<0.001 (S)*	369 (21.1)
Previous treatments within the year before SLIT initiation	*N*	*843*	*907*		*1750*
Oral antihistamines	N (%)	713 (84.6)	822 (90.6)	<0.001 (S)**	1535 (87.7)
Nasal corticoids	N (%)	452 (53.6)	529 (58.3)	0.048 (S)**	981 (56.1)
Asthma medications^a^	N (%)	319 (78.4)	257 (78.1)	0.931 (NS)*	576 (78.3)

*RTSS* rhinitis total symptom score ranging from 0 to 18, *TNSS* total nasal symptom score ranging from 0 to 12

* Chi^2^ Pearson test

** Fisher test (at least one theoretical number ≤5)

^#^ Wilcoxon-Mann–Whitney test (non-Gaussian variable)

^a^At least one long-acting β2-agonist and/or short-acting β2-agonist and/or fixed-dose combination and/or inhaled corticosteroids

Overall, the most common forms of allergy in the participants’ family history were rhinitis (47.3 and 62.4% for adults and children, respectively), conjunctivitis (16.8 and 21.5%) and asthma (27.0 and 40.0%). More than half of participants were polysensitized, most commonly to grass pollen (60.7 and 50.2% for adults and children, respectively), pets (44.4 and 42.7%) and tree pollen (34.3 and 33.3%).

According to the Allergic Rhinitis and its Impact on Asthma (ARIA) 2010 classification [15], participants in the MORPHEE study suffered most commonly from persistent and severe AR (67.3% of adults and 59.4% of children). AR was persistent and mild for approximately a quarter of participants (22.6% of adults and 26.5% of children). However, a small proportion of participants had intermittent rhinitis that was either severe (3.8% of adults and 2.4% of children) or mild (6.3% of adults and 11.7% of children). The mean rhinoconjunctivitis symptom severity (RTSS) score was significantly higher for adults than for children. The same observation was made for rhinitis symptom severity (TNSS).

Adults had been diagnosed with AR for an average of 8.4 ± 9.4 years, and children, for an average of 3.0 ± 3.2 years. In patients with current asthma associated with AR, asthma was most commonly intermittent (48.8%) or mild and persistent (29.3%). According to the Global Initiative for Asthma (GINA) guidelines [16], asthma was controlled in the majority of asthmatic participants (64.4% of adults and 70.3% of children), and 75.5% of participants were in treatment stage 1 or 2.

### Treatment pathways for allergy

Allergy to HDM was most commonly confirmed using a positive skin test (1707 positives out of 1720 tested participants [99.2%]) and/or specific IgE tests (885 positives out of 891 tested participants [99.3%]).

The medications most frequently prescribed within the 12 months prior to enrolment were antihistamines (90.6% of adults and 84.6% of children) and nasal corticosteroids (58.3% of adults and 53.6% of children); 3.1% of adults and 4.0% of children said that they had not received any drug prescriptions in the previous 12 months. According to the 5-item self-administered satisfaction questionnaire, 83% of adults and 75% of children were not satisfied with their current allergy treatment, mainly due to a lack of efficacy in relieving their symptoms (56.6%) and the belief that the quantity of drugs was too large (52.1%).

### Sleep disorders

**Fig. 1:**
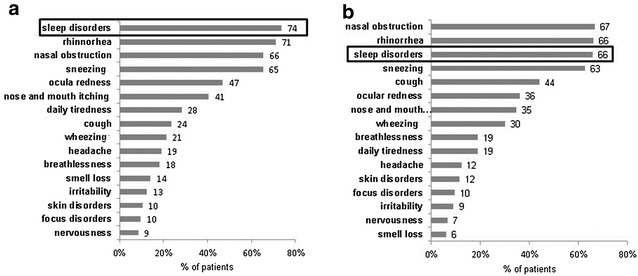
Main symptoms leading to physician visit. The number of participants who mentioned each symptom as a reason for visiting the physician. *Graph* **a** presents data related to adults. *Graph* **b** presents data related to children

The majority of participants (73.5% of adults and 65.8% of children) reported that their sleep problems were the main reason for their consultation, as shown in Fig. 1. The most commonly observed sleep problems were poor-quality sleep (50.3% of adults and 37.3% of children), snoring (48.1 and 41.4%, respectively) and nocturnal awakenings (37.6 and 28.2%). The prevalence rates of sleep complaints/disorders in adults and children are summarized in Tables 3 (adults) and 4 (children). The mean ISI score was 10.1 ± 5.9 for adults and 7.4 ± 5.5 for children (ISI scores from 0-7 refer to no insomnia and ISI scores from 8–14 refer to mild insomnia). The mean ESS score (daytime sleepiness) was 6.7 ± 4.3 for adults and 4.9 ± 3.9 for children (ESS scores from 0–8 indicate no daytime sleepiness).

**Table 3:** Prevalence of sleep complaints/disorders in adults according to type of AR

	Mild intermittent AR n = 51	Severe intermittent AR n = 31	Mild persistent AR n = 184	Severe persistent AR n = 548	OR [95% CI] (severe persistent AR vs. other AR)^e^	Total n = 907^a^
Sleep complaints						
Difficulty falling asleep	7 (13.7)	7 (22.6)	34 (18.6)	171 (31.2)	2.160 [1.458–3.202]^b^	244 (27.0)
Nocturnal awakening	16 (31.4)	7 (22.6)	55 (30.1)	229 (41.8)	1.683 [1.198–2.363]^b^	340 (37.6)
Early awakening	6 (11.8)	2 (6.5)	13 (7.1)	81 (14.8)		117 (12.9)
Poor-quality sleep	19 (37.3)	12 (38.7)	71 (38.8)	307 (56.0)	1.657 [1.200–2.290]^b^	455 (50.3)
Feeling of lack of sleep	20 (44.4)	16 (53.3)	78 (47.9)	325 (64.5)		485 (58.4)
Snoring	17 (33.3)	16 (51.6)	86 (47.0)	272 (49.6)	1.175 [0.848–1.629]^b^	435 (48.1)
ESS >8	10 (21.7)	11 (33.4)	39 (21.3)	171 (30.9)	1.368 [0.935–2.001]^c^	255 (29.7)
Sleep disorders						
Clinical insomnia (i.e., moderate or severe)	8 (15.7)	5 (16.1)	30 (16.5)	176 (32.7)	2.202 [1.475–3.286]^d^	245 (27.4)
Sleep apnea syndrome	6 (11.8)	5 (16.1)	15 (8.2)	73 (13.4)		112 (12.5)
Other						
Regular use of sedatives	2 (4.3)	0	9 (5.1)	35 (6.6)		52 (5.9)

Data are no. (%), unless otherwise specified. Missing data were excluded for percentage calculations

*AR* allergic rhinitis, *OR* odds ratio, *CI* confidence interval

^a^Type of AR was not recorded for 93 participants

^b^Difficulty falling asleep/nocturnal awakening/poor-quality sleep/snoring prediction model: yes vs. no

^c^Daytime sleepiness prediction model: daytime sleepiness (including severe daytime sleepiness) vs. no daytime sleepiness

^d^Insomnia prediction model: moderate or severe insomnia vs. no insomnia or mild insomnia

^e^Multivariable logistic regression

**Table 4:** Prevalence of sleep complaints/disorders in children according to type of AR

	Mild intermittent AR n = 89	Severe intermittent AR n = 18	Mild persistent AR n = 202	Severe persistent AR n = 453	OR [95% CI] (severe persistent AR vs. other AR)^e^	Total n = 843^a^
Sleep complaints						
Difficulty falling asleep	25 (28.4)	1 (5.6)	40 (19.9)	121 (26.8)	1.206 [0.816–1.780]^b^	207 (24.7)
Nocturnal awakening	21 (23.9)	5 (27.8)	51 (25.4)	136 (30.1)	1.142 [0.791–1.648]^b^	237 (28.2)
Early awakening	10 (11.4)	1 (5.6)	19 (9.5)	55 (12.2)		93 (11.1)
Poor-quality sleep	20 (22.7)	4 (22.2)	52 (25.9)	207 (45.8)	2.231 [1.563–3.184]^b^	313 (37.3)
Feeling of lack of sleep	25 (30.9)	4 (25.0)	61 (34.1)	209 (52.5)		325 (43.7)
Snoring	30 (34.1)	7 (38.9)	70 (34.8)	213 (47.1)	1.455 [1.037–2.040]^b^	347 (41.4)
ESS >8	9 (11.6)	1 (6.7)	19 (10.1)	99 (22.5)	2.613 [1.553–4.397]^c^	136 (17.3)
Sleep disorders		1 (5.6)	40 (19.9)	121 (26.8)		
Clinical insomnia (i.e. moderate or severe)	7 (8.0)	1 (6.3)	10 (5.2)	85 (19.6)	3.335 [1.853–6.002]^d^	349 (43.2)
Sleep apnea syndrome	4 (4.5)	0 (0.0)	6 (3.0)	32 (7.1)		47 (5.7)
Other						
Regular use of sedatives	5 (5.9)	0	3 (1.6)	11 (2.5)		22 (2.7)

Data are no. (%), unless otherwise specified. Missing data were excluded for percentage calculations

*AR* allergic rhinitis, *OR* odds ratio, *CI* confidence interval

^a^Type of AR was not recorded for 81 participants

^b^Difficulty falling asleep/nocturnal awakening/poor-quality sleep/snoring prediction model: yes vs. no

^c^Daytime sleepiness prediction model: daytime sleepiness (including severe daytime sleepiness) vs. no daytime sleepiness

^d^Insomnia prediction model: mild or moderate insomnia vs. no insomnia or slight insomnia

^e^Multivariable logistic regression

Logistics regression models adjusted for BMI, smoking status, nasal obstruction and asthma control show that adults suffering from severe persistent AR were more likely to experience difficulties falling asleep (OR 2.160 [1.458–3.202]; p = 0.0001), nocturnal awakenings (OR 1.683 [1.198–2.363]; p = 0.0027), poor-quality sleep (OR 1.657 [1.200–2.290]; p = 0.0022) and clinical insomnia (mainly moderate insomnia OR 2.202 [1.475–3.28]; p = 0.0001). No influence of persistent severe AR on snoring or daytime sleepiness was observed. However, obesity and overweight were shown to be highly related to the prevalence of snoring (ORs 2.728 [1.601–4.647] and 2.136 [1.514–3.014], respectively; p < 0.001) and nasal obstruction was also related to poor-quality sleep (OR 2.018 [1.396–2.917]; p = 0.0002).

Similarly, logistic regression models showed that children with severe persistent AR were more likely to experience poor-quality sleep (OR 2.231 [1.563–3.184]; p < 0.0001), clinical insomnia (OR 3.335 [1.853–6.002]; p < 0.0001), daytime sleepiness (OR 2.613 [1.553–4.397]; p = 0.0003), and snoring (OR 1.455 [1.037–2.040]; p = 0.03), but no effect of severe persistent AR on difficulty falling asleep or nocturnal awakening was observed. Nasal obstruction (moderate or severe) also had an impact on poor-quality sleep (OR 1.575 [1.030–2.407]; p = 0.0359) and snoring (OR 1.683 [1.124–2.521]; p = 0.0115).

Furthermore, both adults and children, who suffered from severe persistent AR were more likely to become uncomfortable with their sleep, particularly regarding disturbances in their daily lives (ORs 2.050 [1.353–3.105] and 3.186 [1.685–6.026], respectively), apparent sleep difficulties (ORs 3.704 [1.883–7.286] and 3.427 [1.760–6.673], respectively), dissatisfaction with their current sleep (ORs 1.802 [1.286–2.525] and 2.900 [1.872–4.494], respectively) and concerns about their difficulties falling asleep (ORs 1.536 [0.923–2.556] and 3.574 [1.561–8.183], respectively).

## Discussion

According to a report published in 2006 by the French Ministry of Health and Solidarity, approximately 20–30% of the French population complained of sleep disorders, 15–20% of whom had moderate insomnia; 9–10%, severe insomnia; and approximately 8%, excessive daytime sleepiness. These conditions each have direct consequences for public health [17]. Many studies have shown that quality of life is frequently affected by respiratory allergies, in particular among patients with moderate to severe symptoms. The related disorders include changes in mood, deterioration in cognitive function and in school and work performance, memory deficits and an inability to concentrate [5, 18–25]. Sleep disruption, sleepiness and tiredness are frequently reported by patients with AR [4, 8, 20, 23, 24, 26]. It has become increasingly clear that sleep disorders have a direct and indirect growing economic impact, and management of sleep disorders thus represents an important challenge for health systems. In our study, sleep disorders were among the most commonly cited reasons for consultation (73.5% of adults and 65.8% of children). For adults, this frequency is twice as high as that obtained in the “Sleep and Transport” yearly survey poll commissioned by the French Institute for Public Health Surveillance (INVS), which reported that 37% of French people suffered from sleep disorders [27]. For participants in the MORPHEE study, the other symptoms that were commonly reported as reasons for consultation were typical of AR [14]: rhinorrhea (71.0% of adults and 66.1% of children), nasal congestion (65.5 and 66.7%), and sneezing (65.3 and 62.8%). In addition, persistent cough was a reason for consultation in 44.1% of children. This finding might be explained by the high frequency of children suffering from asthma (48.3%). Some authors have shown that nasal congestion could be a major cause of sleep disturbance and respiratory disorders during sleep [23, 28]. Other symptoms of rhinitis (sneezing, rhinorrhea, nasal pruritus) and different components of the immune and inflammatory response could also affect sleep and sleepiness during the day [23]. The use of antihistamines was not expected to play a significant role in sleepiness because the participants were almost exclusively taking new-generation antihistamines.

The analysis of the frequency of sleep disorders according to type of AR showed that sleep disorders were more common in severe persistent AR than in other forms of AR. These results support those of the DREAM and SOMNIAAR studies, which previously showed a positive correlation between deterioration in quality of sleep and AR severity [4, 8]. Specifically, the DREAM study showed that participants with severe AR, whether persistent or intermittent, had an increased incidence of insomnia, hypersomnia, sleep apnea, sleepiness and memory problems and a significantly more regular use of sedatives and alcohol compared to those who did not have AR [4]. In the MORPHEE study, participants suffering from severe and persistent AR experienced sleep disorders significantly more frequently than participants in the other groups. This result may be associated with a potential recruitment bias related to the nature of the allergen and the SLIT indications. Indeed, the majority of participants enrolled in the MORPHEE study had severe persistent AR (63.5%), which is a primary indication for the initiation of SLIT. Only 12.0% of participants presented with intermittent AR (mild or severe).

The distribution of participants in the MORPHEE study according to AR category can be compared to those of other studies conducted among participants with AR caused by different allergens (DREAM, SOMNIAAR, ODISSEE and INSTANT studies) or with AR induced by HDM (ADARA and ANTARES studies). In the ADARA study, 75.4% of asthmatic participants (adults and children) and 79.1% of non-asthmatic participants had moderate to severe persistent AR [21]. The ANTARES study showed that 53% of children had moderate to severe persistent AR, with a mean TNSS score of 8.2 ± 2.4; these values were close to those obtained in the MORPHEE study [29]. The high proportion of persistent rhinitis observed in the MORPHEE study (80% of participants) is likely linked to the perennial nature of the allergen (HDM). Indeed, in the DREAM study, sensitization to HDM was observed in 35% of participants with intermittent rhinitis and in 72% of participants with persistent rhinitis, whereas there was no significant difference between participants sensitized to pollen: 87% had intermittent rhinitis and 72% had persistent rhinitis [30]. Therefore, the higher frequency of severe AR in the MORPHEE study may be linked to the recruitment of participants who were candidates for AIT. Indeed, according to the latest ARIA guidelines, AIT is indicated for severe and/or persistent AR that is difficult to control with symptomatic treatments alone or, in the case of rhinitis accompanied by asthma, that justify systemic treatment with AIT [14].

At the time of their consultation, nearly 80% of participants were not satisfied with their treatment before SLIT, mainly because it did not relieve their allergies (56.7%) or because the amount of drugs taken was too high (52.1%). The prescription of AIT is thus consistent with the ARIA good practice guidelines for treating respiratory allergies for patients with moderate to severe AR when symptomatic drugs and avoidance measures cannot relieve their symptoms. Unlike symptomatic treatments, AIT can significantly modify the disease in a clinically relevant way in the years following treatment and in the long term [31, 32].

As the MORPHEE study used a cross-sectional observational design, it had certain limitations. In particular, the method used to document sleep disorders did not allow for assessments of the existence or extent of bias associated with systematic questioning. However, the frequency with which sleep disturbances were reported when questions were asked to both physicians and participants via a self-reported questionnaire clearly demonstrated that sleep disorders are highly prevalent and not always accounted for in the treatment of allergic patients.

This finding highlights the importance of asking patients about the quality of their sleep during allergy consultations to better identify their problems.

Furthermore, the participating physicians may have consciously or unconsciously selected participants for the study; however, this possibility was inevitable. Although the sequential nature of enrolment helped reduce this bias and minimized the potential bias related to the enrolment of more severe participants due to their more frequent consultations, it cannot be ruled out that the participants enrolled in the MORPHEE study tended to have more severe conditions and therefore more intense sleep disorders. Although less robust than clinical trials from a methodological point of view, observational studies do, nonetheless, have the advantage of allowing “real-life” data to be collected and thus reflect routine medical practice when a sufficient number of participants has been included. Finally, the characteristics of the participants in the study were very similar to those of patients with HDM allergies who are usually seen in routine practice; this similarity allowed the results to be generalized to patients starting SLIT for HDM allergy.

## Conclusions

This survey clarified the characteristics and effects of sleep disorders in a large sample of participants consulting their physician for AR caused by HDM who were considering initiating SLIT. The multivariable logistic regression models showed that subjects suffering from severe persistent AR were more likely to experience difficulty falling asleep, nocturnal awakening, clinical insomnia and poor-quality sleep than those with other types of AR.

Consequently, asking patients about the quality of their sleep during consultations for respiratory allergies appears to be important, especially when they have persistent and severe AR.

The results of large placebo-controlled double-blind clinical trials have shown favourable effects of an HDM SLIT tablet, and the results of the MORPHEE survey further encourage exploration of the qualitative and quantitative benefits of SLIT in improving sleep in patients with HDM allergy.

## Abbreviations

AIT: allergen immunotherapy
AR: allergic rhinitis
ARIA: allergic rhinitis and its impact on asthma
BMI: body mass index
CRF: case report form
DSM: diagnostic and statistical manual of mental disorders
ESS: Epworth Sleepiness Scale
GINA: global initiative for asthma
HDM: house dust mite
ICSD: international classification of sleep disorders
ISI: Insomnia Severity Index
RTSS: rhinoconjunctivitis total symptom score
SLIT: sublingual immunotherapy
TNSS: total nasal symptom score
